# Immunomodulatory Effects of Primed Tonsil-Derived Mesenchymal Stem Cells on Atopic Dermatitis via B Cell Regulation

**DOI:** 10.3390/cells13010080

**Published:** 2023-12-30

**Authors:** Dong-Kyu Kim, Hyun-Joo Lee, Il Hwan Lee, Jae-Jun Lee

**Affiliations:** 1 Department of Otorhinolaryngology-Head and Neck Surgery, Chuncheon Sacred Heart Hospital, Hallym University College of Medicine, Chuncheon 24252, Republic of Korea; 2 Institute of New Frontier Research, Division of Big Data and Artificial Intelligence, Chuncheon Sacred Heart Hospital, Hallym University College of Medicine, Chuncheon 24252, Republic of Korea; 3 Department of Anesthesiology and Pain Medicine, College of Medicine, Chuncheon Sacred Heart Hospital, Hallym University College of Medicine, Chuncheon 24252, Republic of Korea

**Keywords:** mesenchymal stem cells, atopic dermatitis, B cells, CD40

## Abstract

Mesenchymal stem cells (MSCs) ameliorate T-and B cell-mediated immune responses. In particular, tonsil-MSCs (T-MSCs) are attractive candidates for practical and clinical applications because of their ease of acquisition and relatively low immunogenicity compared with other MSC sources. The use of MSCs as a therapeutic tool in atopic dermatitis (AD) has been investigated, but that of T-MSCs remains to be explored. Therefore, we investigated the immunomodulatory effects of primed T-MSCs in AD pathogenesis. In our animal study, primed T-MSCs showed greater immunological suppressive effects than naïve T-MSCs. Additionally, in vitro, the proliferation of B cells was downregulated by the addition of primed T-MSCs compared with naïve T-MSCs. The activation of B cells to differentiate into antibody-secreting cells and produce IgE was also reduced when primed T-MSCs were added. Moreover, under CD40-knockdown conditions, we found that CD40 in primed T-MSCs played a critical role as a regulator of B cell activation and was mediated by the non-canonical NF-κB pathway. Therefore, our findings suggest a promising role for primed T-MSCs in the treatment of AD by regulating B cell-mediated inflammatory responses, which are dependent on CD40 expression on primed T-MSCs mediated through the non-canonical NF-κB pathway.

## 1. Introduction

Mesenchymal stem cells (MSCs) have been used in animal studies and clinical trials for decades. MSCs have the potential to differentiate into various mesenchymal cell types and exert immunomodulatory effects. Thus, significant achievements have been made in tissue regeneration and immune disorders using MSCs [1]. MSCs can be isolated from various human tissues including skin, bone marrow, adipose tissue, placenta, and the umbilical cord [2]. Among these, tonsil-MSCs (T-MSCs) can be acquired without unnecessary invasive procedures, because they are commonly harvested from waste tissue after tonsillectomy. Additionally, their relatively high proliferation rates and low immunogenic properties make them attractive sources of MSCs [3,4]. These unique characteristics of T-MSCs can be attributed to their typical isolation from young donors less than 10 years of age, unlike other sources of adult stem cells [5].

Atopic dermatitis (AD) is the most common chronic inflammatory skin disease, and is characterized by intense itching and recurrent eczematous lesions. The incidence and prevalence of AD vary significantly depending on age, with children having a higher prevalence than adults [6]. Clinical symptoms of AD also appear differently in adults and children; however, common features include recurrent dry and severe skin itching. The pathogenesis of AD is multifactorial and includes genetic predisposition, epidermal barrier disruption, and immune system involvement [7]. Various studies have demonstrated that type 2 immune cytokines, including interleukin-4 (IL-4), IL-13, IL-17, IL-22, IL-31, and thymic stromal lymphopoietin, play important roles in the pathogenesis of AD [8,9,10]. Th2 cells induce IgE class switching in B cells, resulting in increased IgE levels frequently observed in patients with AD [11]. B cells also contribute to antigen-specific Th2 cell activation and expansion during allergic skin inflammation. Therefore, antigen-specific B cells are essential for Th2 cell development [12]. The treatment of AD focuses on controlling disease progression and alleviating symptoms, because there is currently no cure for or prevention for AD. Thus, the treatment of AD targets underlying skin abnormalities such as xerosis, pruritus, and cutaneous inflammation [13].

To date, several studies have demonstrated that MSCs derived from various tissues, such as bone marrow and adipose tissue, exert immunomodulatory effects in AD [14,15]. However, whether allergic progression in AD can be suppressed by T-MSCs has not yet been clearly defined. Therefore, this study aims to explore the potential immunomodulatory effects of T-MSCs on the pathogenesis of AD in vivo and in vitro.

## 2. Materials and Methods

### 2.1. Isolation and Characterization of T-MSCs

T-MSC isolation was approved by the Hallym University Hospital Institutional Review Board, and written informed consent was obtained from healthy donors. T-MSCs were isolated from tonsillar tissue as described previously [16]. T-MSCs were characterized using the following antibodies: anti-CD31 (BioLegend, San Diego, CA, USA), anti-CD44 (BD Biosciences, San Diego, CA, USA), anti-CD45 (BioLegend), anti-CD105 (BioLegend), anti-CD73 (Biogems, Westlake Village, CA), anti-CD80 (BioLegend), anti-CD86 (BioLegend), anti-HLA class I (BioLegend), anti-HLA-DR (BioLegend), and anti-CD275 (BioLegend). Cell surface antigens were analyzed by flow cytometry (FACSCalibur or FACScanto II; BD Biosciences). Isotype-matched control antibodies were used as controls. The T-MSCs used in this study exhibited the typical phenotype.

### 2.2. Isolation and Stimulation of B Cells

To investigate whether primed T-MSCs directly regulated the activation of B cells, T-MSCs were treated with 10 ng/mL tumor necrosis factor-α (TNF-α) and 20 ng/mL interferon-γ (IFN-γ) for 24 h. We used tonsillar mononuclear cells (TMCs) containing B cell follicles obtained from pediatric tonsillectomy. Tonsillar tissues were dissected, cut into small tissue pieces, and treated with collagenase type I and DNase I (Sigma-Aldrich, St. Louis, MO, USA) for 30 min at 37 °C. This digested tissue was filtered through a 70 µm cell strainer and subjected to Ficoll-Paque (GE Healthcare, Little Chalfont, UK) density gradient centrifugation, following which TMCs were isolated. For B cell-activating conditions, the isolated TMCs (1 × 106 cells/mL) were co-cultured with T-MSCs or primed T-MSCs (1 × 105 cells/mL) in a 24-well plate and stimulated with 0.5 µg/mL of soluble CD40L (BioLegend), 1 µg/mL of CpG oligodeoxynucleotides (ODN 2006, Invivogen, San Diego, CA, USA), 20 ng/mL of recombinant human IL-2 (BioLegend), and 20 ng/mL of recombinant human IL-4 (BioLegend) for 5 days. For some experiments, B cells were isolated using the MojoSort Human Pan B Cell Isolation Kit (BioLegend), and CD4+ T cells were isolated from TMCs using a negative CD4+ T cell isolation kit (Miltenyi Biotec, Bergisch-Gladbach, Germany). Purified human CD4+ cells were polarized using a human Th2 cell differentiation kit (#CDK002, R&D Systems) at a density of 5.0 × 105 cells/mL. After 3 days, 5.0 × 105 cells/mL of B cells were co-cultured in a 24-well microplate (1 mL/well) at 5% CO_2_ at 37 °C for 3 d.

### 2.3. Cell Proliferation Assay

To examine the effect of primed T-MSCs on stimulated B cell proliferation, TMCs were labeled with carboxyfluorescein diacetate succinimidyl estercarboxyfluorescein diacetate succinimidyl ester (CFSE; Invitrogen, Waltham, MA, USA) according to the manufacturer’s instructions. The labeled cells were co-cultured with T-MSCs or primed T-MSCs for 5 d. After co-culture, flow cytometric analysis of cell division by CFSE dilution was conducted to assess B cell proliferation.

### 2.4. Real-Time PCR (RT-PCR)

To evaluate the mRNA expression of transcription factors related to B cell differentiation, we used CD19+ B cells isolated from TMCs co-cultured with T-MSCs or primed T-MSCs under B cell-activating conditions. CD19+ B cells were obtained using the Mojosort Human B Cell Isolation Kit (BioLegend) according to the manufacturer’s instructions. Also, to confirm the mRNA expression of IL-4 and IL-13 in skin lesions of the atopic dermatitis model, total RNAs were extracted from dorsal skin tissues. Total RNA was isolated using the easy-BLUE reagent (Intron Biotechnology, Seongnam, South Korea) according to the manufacturer’s recommendations. cDNA was synthesized from 2 μg of total RNA using an AccuPower cDNA Synthesis Kit (Bioneer, Daejeon, South Korea). The cDNA was then amplified and quantified using the SYBR Green master mix (Applied Biosystems, Foster City, CA, USA) with the following primers: human β-actin, forward 5′-GTG CTA TCC CTG TAC GCC TC-3′ and reverse 5′-GGC CAT CTC TTG CTCGAAGT-3′; human Blimp-1, forward 5′-TAC ATA CCA AAG GGC ACA CG-3′ and reverse 5′-TGA AGC TCC CCT CTG GAA TA-3′; human XBP-1, forward 5′-CCT GGT TGC TGA AGA GGA GG-3′ and reverse 5′-CCA TGG GGA GAT GTT CTG GAG -3′; human BCL-6, forward 5′- GAG AAG CCC TAT CCC TGT GA-3′ and reverse 5′-TGC ACC TTG GTG TTG GTG AT- 3′, mouse GAPDH, forward 5′-ACC ACA GTC CAT GCC ATC AC-3′ and reverse 5′-TGG ACC ACC CTG TTG CTG TA-3′; mouse IL-4, forward 5′- ACA GGA GAA GGG ACG CCA T′ and reverse 5′- GAA GCC CTA CAG ACG AGC TCA -3′; mouse IL-13, forward 5′- TCT TGC TTG CCT TGG TGG TCT CGC -3′ and reverse 5′- GAT GGC ATT GCA ATT GGA GAT GTT G -3′.

### 2.5. Western Blotting

To determine the signaling pathways affected by priming with TNF-α and IFN-γ, T-MSCs were pretreated with the signaling pathway inhibitor, AMGEN16 (non-canonical NF-κB pathway, 5 μmol/L; Sigma-Aldrich). After pretreatment with an inhibitor, the cells were incubated with TNF-α and IFN-γ for 24 h and then lysed in RIPA buffer. The lysates were separated using 10% sodium dodecyl sulfate-polyacrylamide gel electrophoresis and were transferred onto polyvinylidene fluoride membranes (Millipore, Billerica, MA, USA). The membranes were blocked with blocking solution and incubated with antibodies against phospho-p65 (#3033, Cell signaling Technology, Danvers, MA, USA), p65 (#8242, Cell signaling) of the canonical NF-κB pathway, and phospho-p100 (#4810, Cell signaling), p52 (#4882, Cell signaling), and RELB (#4922, Cell signaling) of the non-canonical NF-κB pathway (Cell signaling Technology, Danvers, MA, USA), an-ti-CD40 (# ab224639, abcam), and β-actin (#4970, Cell signaling Technology). The blots were visualized using horseradish peroxidase-conjugated secondary antibodies and an enhanced chemiluminescence (ECL) system (Pierce, Rockford, IL, USA).

### 2.6. Flow Cytometry

B cell activation was analyzed by detecting surface or intracellular markers using flow cytometry. Prior to staining for surface markers, dead cells were excluded by staining with LIVE/DEAD-fixable violet dye (Invitrogen). To examine the phenotype of antibody-secreting cells (ASCs) in B cell activation, the Fc receptors (FcR) were blocked using an FcR blocking reagent (Miltenyi Biotec, Bisley, Surrey, UK) and stained with allophycocyanin (APC) or phycoerythrin (PE) anti-human CD19 antibody (BioLegend), fluorescein isothiocyanate (FITC)-conjugated anti-human CD27 antibody (BioLegend), and APC/Cyanine7anti-human CD38 antibody (Biolegend). To detect IgE production by B cells, the cells were incubated with a fixation/permeabilization solution (eBioscience, San Diego, CA, USA) and stained with PE/Cy7 anti-IgE (BioLegend). After staining, the cells were analyzed using FACSCalibur or FACSCanto Ⅱ (Becton Dickinson, CA, USA), and the data were evaluated using BD FlowJo (BD Bioscience, La Jolla, CA).

### 2.7. SiRNA Transfection

T-MSCs were transfected with 40 pmol siRNA against human CD40 (sc-29250; Santa Cruz Biotechnology, Santa Cruz, CA, USA) or control siRNA (sc-37007; Santa Cruz Biotechnology) using siRNA transfection reagent (Santa Cruz Biotechnology). Twenty-four hours after transfection, the cells were primed with TNF-α and IFN-γ for 24 h, and the expression level of CD40 was analyzed. Primed T-MSCs with downregulated CD40 expression were co-cultured with TMCs under B cell-activating conditions.

### 2.8. Immunofluorescence

After the indicated treatments, T-MSCs were fixed with 4% paraformaldehyde (PFA) at room temperature for 10 min, permeabilized by incubation with a 0.05% Triton X-100 solution for 10 min, and blocked with 5% normal goat serum for 1 h. The cells were subsequently stained with specific primary antibodies against anti-CD40 (1:200; Abcam, Cambridge, MA, USA) and anti-RELB (1:100; Cell Signaling Technology, Danvers, MA, USA), followed by 2 h of incubation with Alexa 488 and Alexa 594-labeled secondary antibodies (1:1000; Molecular Probes, Eugene, OR, USA). For counterstaining, the nuclei were stained with DAPI. Images were captured using a confocal microscope (Carl Zeiss, Jena, Germany).

### 2.9. Mouse Model of AD

To evaluate whether primed T-MSCs affect the development of atopy in vivo, we used a 2,4-dinitrofluorobenzene (DNFB; Sigma-Aldrich, st. louis MO USA)-induced animal model of AD. Female C57BL/6J mice (6-weeks-old, *n* = 24) were divided into the following four groups (*n* = 6 per group): untreated mice (control), DNFB-sensitized and PBS-treated mice (DNFB-only group), DNFB-sensitized and T-MSC-treated mice (T-MSC group), and DNFB-sensitized and primed T-MSC-treated mice (primed T-MSC group). The mice received cutaneous administration of 25 μL of 0.15% DNFB in acetone/olive oil (AO) at a ratio of 3:1 into the right ears, and 100 μL 0.15% DNFB in AO was applied to the shaved dorsal skin on days 0 and 4. Sensitized mice were challenged by applying 0.2% DNFB in AO to the dorsal and ear skin on days 7, 9, and 11, and PBS, T-MSCs, or primed T-MSCs (1 × 10^6^ cells) were injected subcutaneously on days 10 and 12. In this study, we used Matrigel^®^ (MA, BD Biosciences, San Jose, CA, USA) as a scaffold for stem cells. Thus, T-MSCs in 20 μL of PBS were mixed with Matrigel^®^ at a ratio of 1:10 to produce a final volume of 200 μL. We also used a PBS control group by combining 20 μL of PBS with 180 μL of Matrigel^®^. Next, the Matrigel^®^-embedded T-MSCs was subcutaneously administered to the dorsal skin using a 26-gauge sterile needle syringe. Thereafter, the mice were sacrificed, and the dorsal skin and serum were harvested for further examination. The severity of dermatitis was assessed based on the following symptoms: (1) edema, (2) erythema/hemorrhage, (3) dryness/scarring, (4) pruritus/itching, and (5) excoriation/erosion. Symptom scores were expressed as zero (none), one (mild), two (moderate), or three (severe), and each symptom score was assessed by two independent researchers and summed. To evaluate the histological events in AD mice, the dorsal skin was collected, fixed in 10% neutral formalin for 24 h, and embedded in white paraffin. The block was cut into 5 μm-thick sections, and the sectioned slides were stained with hematoxylin-eosin (H&E) or toluidine blue to analyze epidermis thickness and mast cell infiltration. To examine the localization of T and B cells in the skin, slides were stained with anti-mouse CD3 monoclonal antibody (BioLegend) and anti-mouse B220 monoclonal antibody (BioLegend). In this study, we detected the primary anti-mouse CD3 and B220 monoclonal antibodies with horseradish peroxidase-conjugated (HRP)-linked secondary antibody (goat anti-rabbit IgG, 1:1000, Santa Cruz). Then, HRP activity was detected with 3,3′-diaminobenzidine (DAB, Roche, Basel, Switzerland) substrate. Slides were imaged using microscopy (Carl Zeiss, Jena, Germany), and six different regions were analyzed using Image J software (Version 1.8.0). Moreover, IL-4 and IL-13 mRNA expression were assessed using RT-PCR in skin tissues.

### 2.10. IgE Detection

On day 24, whole blood samples were collected from the mice by removing the eyeballs after anesthesia. The blood was allowed to clot at 25 °C for 30 min, centrifuged at 1000× *g* for 10 min at 4 °C, and serum was collected and preserved at −80 °C until use. Serum IgE was detected using a mouse IgE enzyme-linked immunosorbent assay kit (#88-50460-22, Invitrogen) according to the manufacturer’s instructions.

### 2.11. Cell Viability Assay

To examine the cytotoxicity of NIKi, cell viability was evaluated using the CCK-8 assay. T-MSCs were seeded at a density of 1 × 104 cells per well in 96-well plates and cultured for 24 h. Then, cells were stimulated with various concentrations of NIKi with or without TNF-α and IFN-γ stimulation and incubated for 24 h. After incubation,10 μL of CCK-8 solution (Dojindo Laboratories, Kumamoto, Japan) was treated to each well for 2 h at 37 °C, and cell viability was measured using a microplate reader (Glomax; Promega, Seoul, Korea) at a wavelength of 450 nm.

### 2.12. Statistical Analysis

All data are presented as the mean ± standard error of the mean. Statistical differences were assessed by Student’s t-test or one-way ANOVA using GraphPad Prism (version 5; Graph Pad Software, San Diego, CA, USA). The statistical values are detailed in the figure legend.

## 3. Results

### 3.1. Effect of Primed T-MSCs on the AD Mouse Model

**Figure 1:**
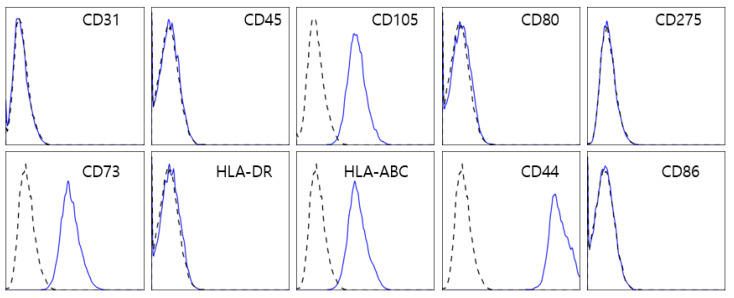
Flow cytometry analysis of T-MSCs. T-MSCs isolated from the human palatine tonsillar tissue were positive for typical MSC markers, including CD105, CD73, CD44, and human leucocyte antigen (HLA) class I, and negative for not only CD31, CD45, and HLA-DR but also co-stimulatory molecules such as CD80 and CD86.

In this study, we used T-MSCs isolated from human palatine tonsillar tissues. To characterize the profile of T-MSCs, we performed flow cytometry (Figure 1). The cells were positively labeled with human MSC markers such as CD44, CD73, CD105, and HLA-Class I, but negative for hematopoietic antigens (CD45 and HLA-DR), endothelial cell markers (CD31), and co-stimulatory molecules (CD80 and CD86).

**Figure 2:**
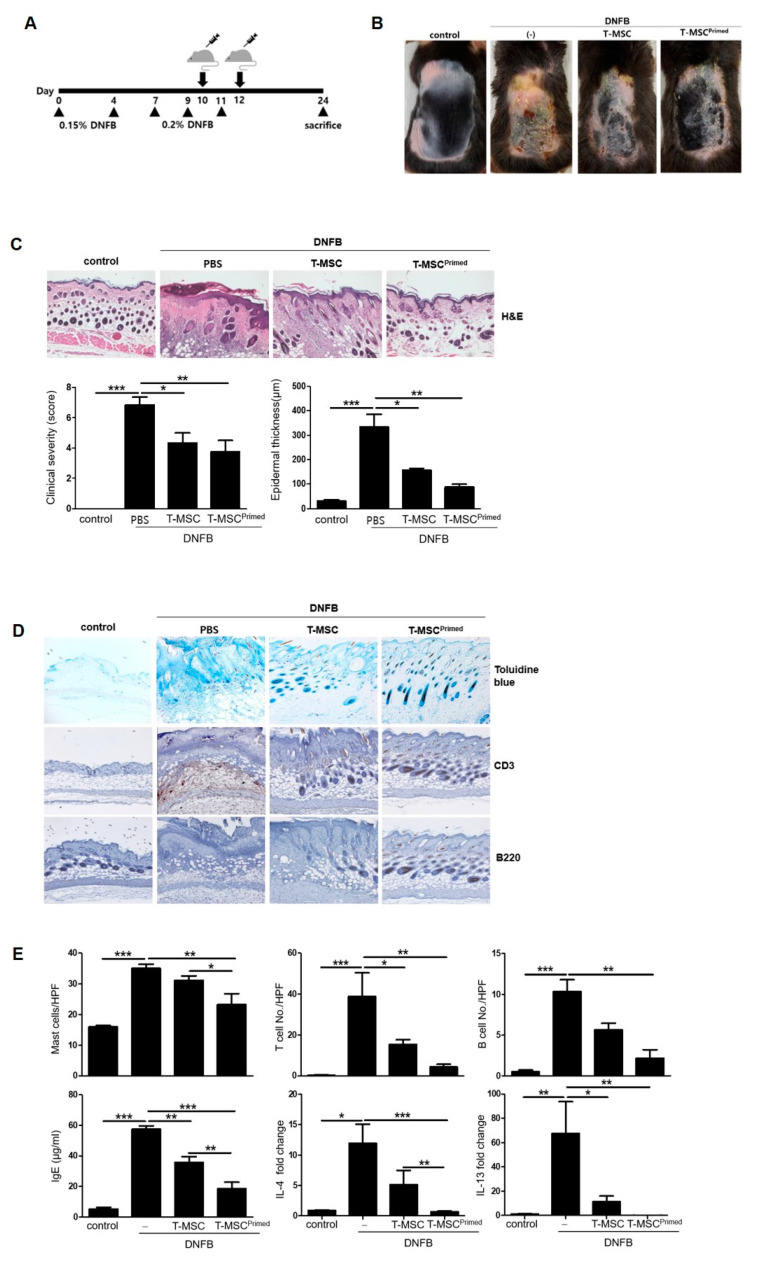
Primed T-MSCs alleviated intense symptoms in the atopic dermatitis mouse model. (**A**) The design scheme of the atopic dermatitis (AD) model. The mice were immunized with 0.15% DNFB (2,4-Dinitrofluorobenzene) on days 0 and 4 and challenged with 0.2% DNFB on days 7, 9, and 11. T-MSCs were subcutaneously injected two times on days 8 and 10 during the sensitization procedures. Each group of mice was monitored and sacrificed on day 24 for further ex vivo examinations. (**B**) Representative gross images of dorsal area lesions on the mice. (**C**) Histopathological examination of skin as assessed by hematoxylin and eosin (H&E) staining. Clinical severity was assessed by scoring dryness, excoriation, erythema, edema, and epidermal thickness in H&E-stained sections. (**D**) Representative histology image by toluidine blue staining for mast cell infiltration and by immunohistochemistry for T cells and B cells in mouse skin. (**E**) Cell counts for mast cell, T cell, and B cell. Serum Ig E levels detected by ELISA. IL-4 and IL-13 mRNA expression in skin lesions. Control: negative control group, DNFB: DNFB-induced AD group, T-MSC: T-MSC-treated group, T-MSC primed: primed-T-MSC-treated group. HPF, high-powered field (×400). Three independent experiments were performed. The results are shown as the mean ± SD. * *p* < 0.05, ** *p* < 0.01, *** *p*< 0.001.

To examine whether the priming effect of IFN-γ and TNF-α can enhance the therapeutic effect of T-MSCs in vivo, we used a DNFB-induced murine model of AD (Figure 2A). We found that the T-MSCs and primed T-MSC groups alleviated the severity of the atopic lesions and decreased the epidermal thickness compared to the DNFB-only groups. Additionally, clinical severity and epidermal thickness scores were significantly lower in the primed T-MSC group than in the T-MSC group (Figure 2B,C). Specifically, the dorsal skin of mice treated with primed T-MSCs showed remarkably improved healing of skin tissue compared to that of DNFB-only mice, in terms of entire epithelium destruction and the extended dermis to infiltrate inflammatory cells.

Next, we evaluated the number of infiltrated mast cells, T cells, and B cells in the dorsal skin in the groups, and assessed the expression of IgE, IL-4, and IL-13 (Figure 2D,E). The cells were identified using toluidine blue and anti-CD3 and anti-B220 antibodies. The dorsal skin of the primed T-MSC-treated group exhibited the lowest number of infiltrated mast, T, and B cells among all the groups. Moreover, we found that the primed T-MSC group showed significantly decreased serum IgE expression and downregulated IL-4 and IL-13 mRNA expression in skin tissue.

### 3.2. Effect of Primed T-MSCs on the Activation of B Cells

**Figure 3:**
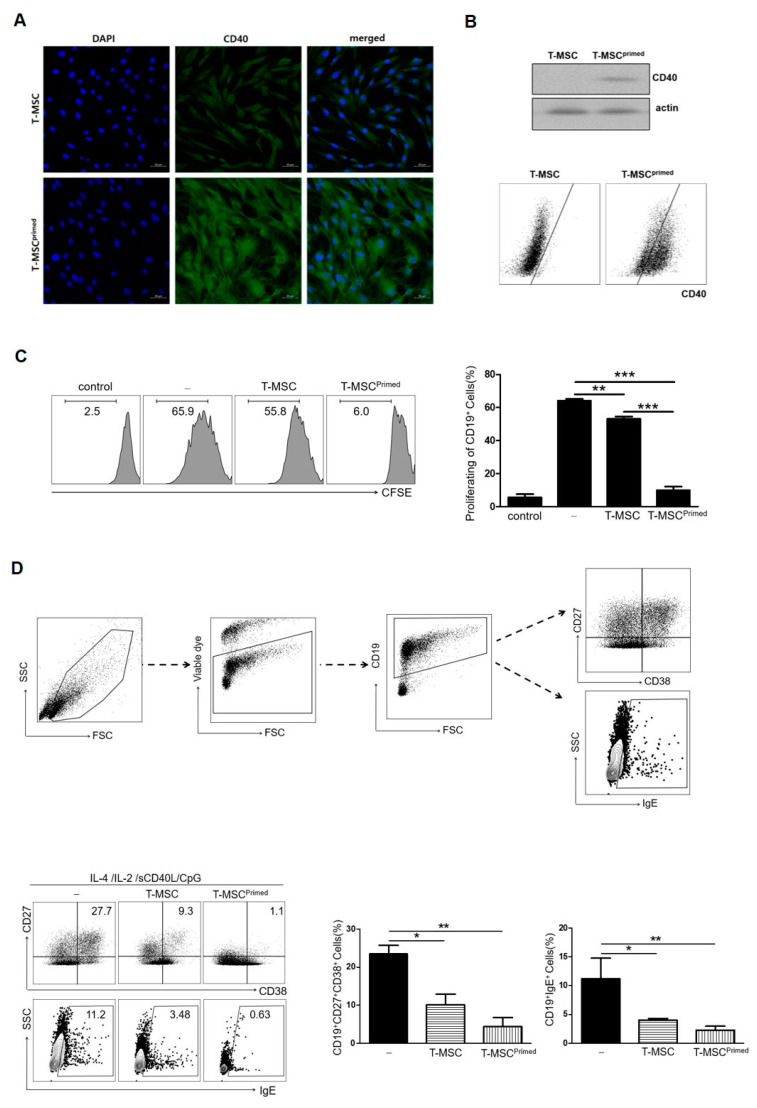
Primed T-MSCs inhibited the activation of B cells. (**A**) Immunofluorescence staining of CD40 expression in control and primed T-MSCs. DAPI was used for nuclear staining. (**B**) Western blot and flow cytometry were used to assess CD40 expression in control and primed T-MSCs. (**C**) To assess B cell proliferation, TMCs isolated from healthy donors were co-cultured for 5 d without T-MSCs, with T-MSCs, and with primed T-MSCs suspensions under B cell-activating conditions (CpG 2006, rCD40L, IL-2, IL-4): (-), non T-MSC group; T-MSC, T-MSC-treated group; T-MSC^primed^, primed T-MSC- treated group. Proliferation was assessed using CFSE. (**D**) Flow cytometry analysis was conducted to examine the phenotype of antibody-secreting cells and IgE production of B cells according to the TMC without T-MSCs, with T-MSCs, and with primed T-MSCs: (-), non T-MSC group; T-MSC, T-MSC-treated group; T-MSC^primed^, primed T-MSC-treated group. In vitro experiments were conducted in triplicates. The results are shown as the mean ± SD. * *p* < 0.05, ** *p* < 0.01, *** *p*< 0.001.

According to our previous study [16], priming T-MSCs considerably increased CD40 expression. In this study, we confirmed this phenomenon (Figure 3A,B) and investigated the mechanisms by which primed T-MSCs expressing CD40 exerted their therapeutic effects on atopic inflammation. In the in vivo study, we found that compared to T-MSCs, primed T-MSCs could more efficiently suppress serum IgE and B cell infiltration, and this efficacy can be attributed to the systemic regulation of allergic responses. Thus, we examined whether primed T-MSCs could suppress B cell inflammation in vitro because these cells are one of the main contributors to the development of atopic inflammation.

First, we examined B cell proliferation to test the suppressive ability of primed T-MSCs. TMCs were co-cultured with T-MSCs or primed T-MSCs for 5 d under B cell-activating conditions (the presence of CpG 2006, rCD40L, IL-2, and IL-4). The proliferation of B cells was significantly inhibited by co-culture with primed T-MSCs compared to T-MSCs (Figure 3C). Second, we examined the effect of primed T-MSCs on the activation of B cells to differentiate into antibody-secreting cells (ASCs) and produce IgE. To analyze the ASC phenotype and IgE production, dead cells were excluded by staining with viability markers, and B cells in TMCs were identified by assessing the expression of CD27, CD38, and IgE in CD19-positive cells (Figure 3D). After 5 d of co-culture, ASCs markedly decreased, and the proportion of B cells producing IgE was significantly reduced in the co-culture with primed T-MSCs compared to T-MSCs (Figure 3D).

### 3.3. Comparison of the Suppressive Function of B Cell Activation by Primed T-MSCs between Direct (Cell–Cell Contact) and Indirect (Transwell) Interactions

**Figure 4:**
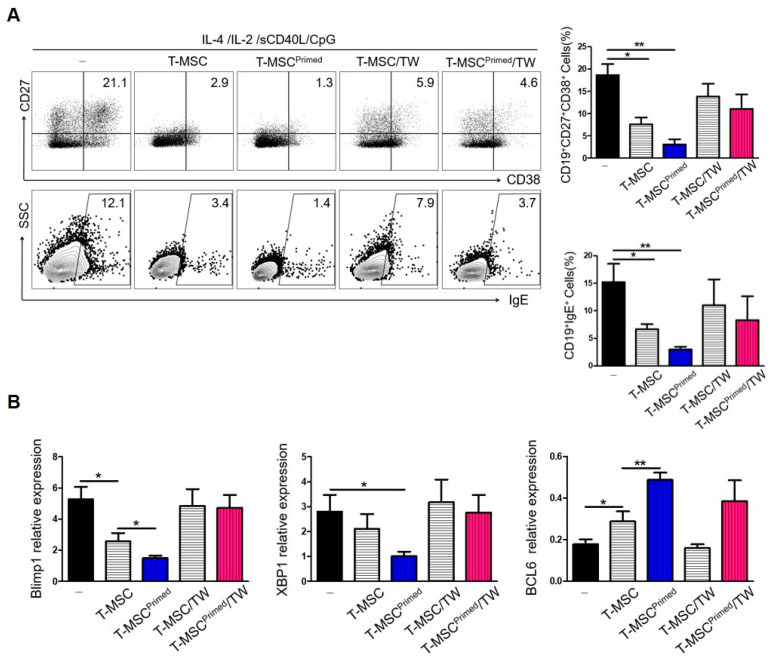
Suppression of B cell activation by primed T-MSCs requires direct cell contact: (-), non T-MSC group. (**A**) To separate TMCs from T-MSCs in B cell-activating conditions, TMCs were co-cultured with T-MSC or T-MSC primed in 24-well transwell plates with a 0.4-mm pore membrane for 5 d. The phenotype of ASCs and IgE production of B cells was analyzed by FACS. (**B**) RNA was isolated from B cells of TMCs co-cultured for 5 d under B cell-activating conditions. The mRNA expression of Blimp-1, XBP-1, and BCL-6 was analyzed by real-time PCR. In vitro experiments were conducted in triplicates. The results are shown as the mean ± SD. * *p* < 0.05, ** *p* < 0.01.

We assessed whether cell contact (i.e., surface molecules such as CD40) or soluble factor-dependent mechanisms could mediate the effects of T-MSCs and primed T-MSCs on B cell activation. When direct cell contact was prevented, the suppressive effect of T-MSCs or primed T-MSCs on B cells in terms of ASC differentiation and IgE production from B cells was abolished (Figure 4A).

Next, to examine whether the suppression of IgE production and the ASC phenotype were related to the regulation of transcription factors involved in plasma cell differentiation by T-MSCs, the expression levels of Blimp-1, XBP-1, and BCL-6 mRNA were evaluated. Under cell-contact-dependent conditions, the mRNA levels of BCL-6 were decreased, while those of XBP-1 and BLIMP-1 were increased (Figure 4B). These findings indicate that B cells were in the process of class switch recombination and differentiation into plasma cells. Collectively, these results suggest that primed T-MSCs activated B cells by regulating BLIMP-1, XBP-1, and BCL6 expression in B cells in a contact-dependent manner.

### 3.4. Regulation of B Cells by Primed T-MSCs Is CD40-Dependent

**Figure 5:**
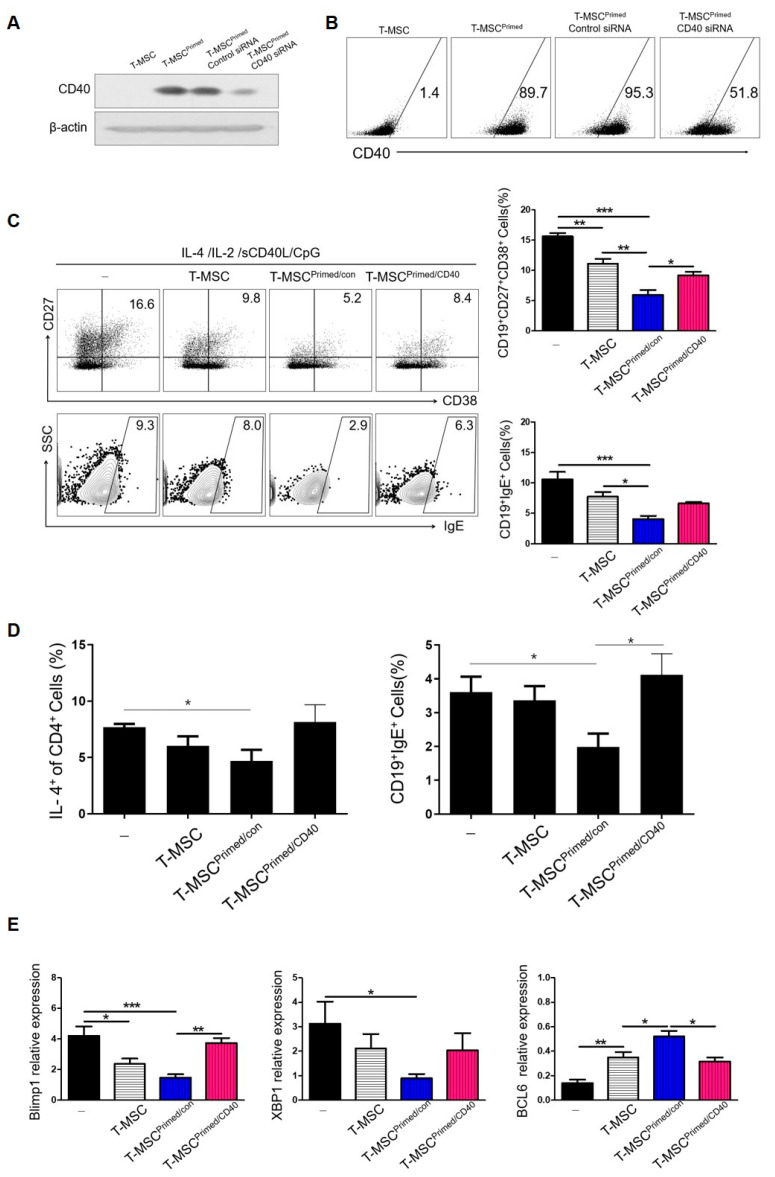
Regulatory effects of B cells by primed T-MSCs were dependent on CD40: (-), non T-MSC group. (**A**,**B**) T-MSCs were transfected with control siRNA (siCon) or CD40-specific siRNA (siCD40). Twenty-four hours after transfection, CD40-knockdown primed T-MSCs were assessed by western blotting and flow cytometry. (**C**) TMCs were co-cultured without T-MSCs or with T-MSCs or with primed-control T-MSC (T-MSCprimed/Con) or with CD40-knockdown primed T-MSCs (T-MSCprimed/CD40) in B cell-activating conditions for 5 d, and IgE production and ASC phenotype of B cells were analyzed by flow cytometry. (**D**) The regulatory effect of primed T-MSC on Th2-mediated B cell activation gating strategy. (**E**) B cells were isolated from TMCs co-cultured without T-MSCs or with T-MSCs or T-MSCsiCon or T-MSCsiCD40 for 5 d in B cell activating conditions, and the mRNA expression of Blimp-1, XBP-1, and BCL-6 in the isolated B cells was analyzed by real-time PCR. In vitro experiments were conducted as triplicates. The results are shown as the mean ± SD. * *p* < 0.05, ** *p* < 0.01, *** *p* < 0.001.

To investigate the role of CD40 as a regulator of B cell activation by primed T-MSCs, we knocked down CD40 using CD40-specific siRNA. Western blotting and FACS analyses confirmed that the expression of CD40 was substantially decreased in primed T-MSCs after CD40 knockdown (Figure 5A,B). Under B cell-activating conditions, compared to primed T-MSCs transfected with control siRNA, CD40-knockdown primed T-MSCs significantly increased the induction of ASCs and IgE production in TMC (Figure 5C).

To further clarify the inhibitory effect of CD40 expression of T-MSCs on IgE production, we tested the effect of primed T-MSCs on Th2-mediated B cell activation. Some studies have reported that T cells are required for the inhibition of B cell activity by MSCs. CD4+ T cells were polarized for 3 d using a Th2 differentiation kit (R&D Systems, Minneapolis, MN, USA) and co-cultured with B cells at a ratio of 1:1 in the presence or absence of T-MSCs. Primed T-MSCs significantly inhibited the proportion of ASCs and IgE expression, and CD40 knockdown primed T-MSCs reversed the suppressive effect on B cell activation (Figure 5D).

**Figure 6:**
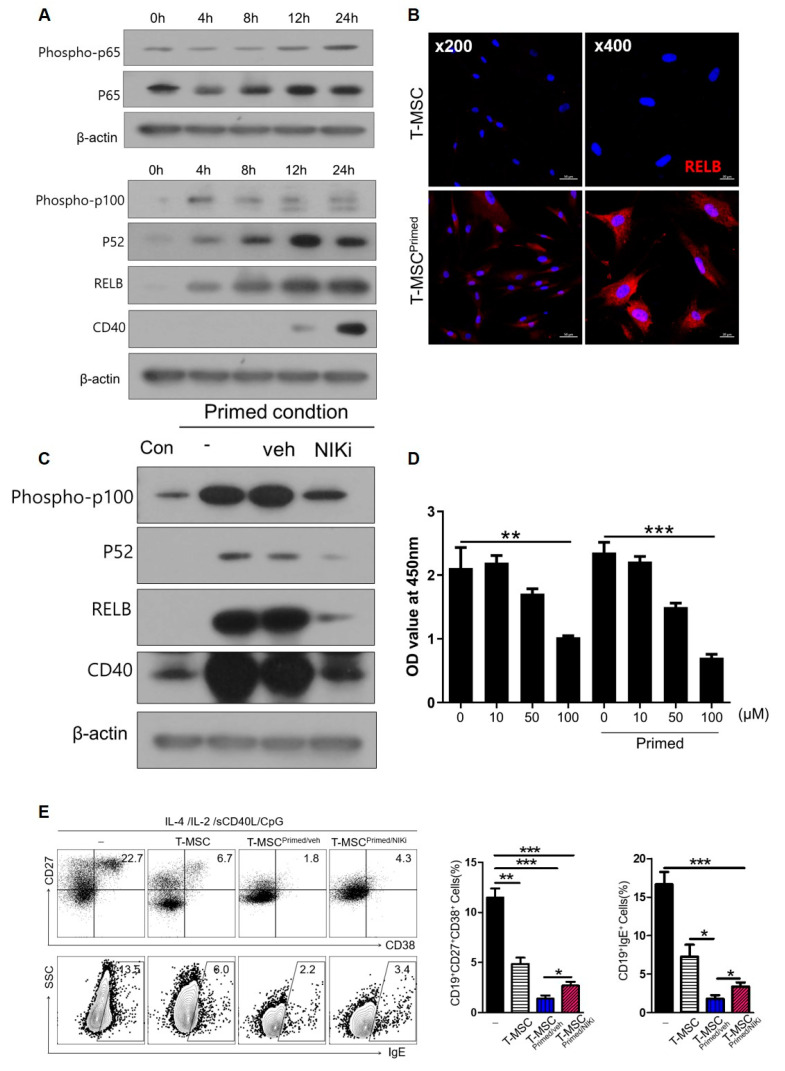
Non-canonical NF-κB pathway in T-MSCs upregulated CD40 expression: (-), non T-MSC group. (**A**) Western blot analysis of non-canonical and canonical NF-κB pathway in T-MSCs primed with TNF-α/IFN-γ. (**B**) Immunofluorescence staining of RELB expression in control and primed T-MSCs. DAPI was used for nuclear staining. (**C**) Western blot analysis of inhibition of the non-canonical pathway of NF-κB using NIK inhibitor (NIKi) in T-MSCs primed with TNF-α/IFN-γ. (**D**) CCK-8 assay: Effect of NIKi at different concentrations on T-MSCs. (**E**) In stimulating B cells, TMCs were cultured without T-MSC or co-cultured with T-MSCs or T-MSCprimed/veh or T-MSCprimed/NIKi for 5 d, and analyzed for IgE production and ASC phenotype of B cells by flow cytometry. Data are shown as the mean ± SD. * *p* < 0.05, ** *p* < 0.01, *** *p*< 0.001.

Moreover, TMCs regulated by CD40-knockdown primed T-MSCs exhibited restored expression of Blimp-1, XBP-1, and BCL6 mRNAs (Figure 6E). Collectively, these data indicate that primed T-MSCs suppress B cell activation in a CD40 expression-dependent manner.

### 3.5. CD40 on Primed T-MSCs Regulates B Cell Activation Via the Non-Canonical NF-κB Pathway

NF-κB signaling is involved in cytokine-induced co-stimulatory molecules on MSCs. In particular, IFN-γ/TNF-α, used as a priming reagent in this study, is a major activator of the NF-κB pathway. To examine the mechanism by which priming by IFN-γ/TNF-α induces CD40 expression, we assessed the non-canonical and canonical NF-κB pathways. After priming with IFN-γ/TNF-α, we observed time-related changes in p100, p52, RELB of the non-canonical NF-κB, and phospho-p65, p65 of the canonical NF-κB. We also found that the non-canonical NF-κB was activated more dramatically than the canonical NF-κB pathways in T-MSCs (Figure 6A). We detected a time-dependent increase in factors related to the non-canonical NF-κB pathway, and CD40 expression was also induced after 24 h. The non-canonical NF-κB pathway predominantly targets activation of the p52/RELB NF-κB complex. Thus, we confirmed increased RELB expression by immunofluorescence staining (Figure 6B). We then investigated whether CD40 expression in primed T-MSCs was regulated by the non-canonical NF-κB pathway. To this end, we inhibited the non-canonical pathway of NF-κB using NIK inhibitor (NIKi). Western blot analysis revealed that the protein levels of p100, p52, RELB, and CD 40 decreased after NIKi treatment (Figure 6C). We then analyzed the role of non-canonical NF-κB signaling activation in primed T-MSC-mediated immunomodulation. We primed T-MSCs by treating them with IFN-γ/TNF-α in the presence and absence of NIKi. Before this investigation, we assessed cell viability via CCK-8 assay to determine the effect of NIKi concentration on T-MSCs (Figure 6D) and then co-cultured TMCs without T-MSC or with T-MSCs or primed T-MSC or primed T-MSC with NIKi under B cell-activating conditions for 5 d. Our findings revealed that NIKi-treated primed T-MSCs significantly reversed the ASC phenotype and IgE production compared with the control (vehicle-treated primed T-MSCs) (Figure 6E). Thus, our findings suggest that CD40 expressed on primed T-MSCs through the non-canonical NF-κB pathway may regulate B cell activation.

## 4. Discussion

To our knowledge, this study is the first to investigate the immunomodulatory effects of primed T-MSCs on AD and B cell regulation. In the animal study, we found that primed T-MSCs exhibited greater therapeutic effects than naïve T-MSCs by inhibiting immune cell infiltration, IgE production, and cytokine expression in a mouse model of AD. In the in vitro study, the proportion of B cells producing IgE was significantly reduced when co-cultured with primed T-MSC with highly expressed CD40 than with T-MSCs. Additionally, we demonstrated that primed T-MSCs activated B cells by regulating BLIMP-1, XBP-1, and BCL6 expression in a contact-dependent manner. Moreover, to investigate the function of CD40 as a regulator of B cell activation by primed T-MSCs, we knocked down CD40 using CD40-specific siRNA. We found that the suppression of B cell activation by primed T-MSCs was dependent on CD40 expression and was mediated by the non-canonical NF-κB pathway.

T and B cells play important roles as immunologic factors in the pathogenesis of AD [17]. Th2 cell activation and related cytokines lead to B cell activation, which elevates the production of allergen-specific IgE, increases skin inflammation, and aggravates skin barrier defects. One study demonstrated that bone marrow-derived MSCs suppressed the Th2 immune response in a mouse model of AD by inhibiting IL-4 and IgE production [14]. Another study showed that adipose tissue-derived MSCs can alleviate AD in a mouse model of AD through regulating B cell-mediated IgE production [15]. Umbilical cord blood-derived MSCs also decreased atopic inflammation in an animal model by regulating mast cell function [18]. Another study showed that T-MSC injection in an AD mouse model reduced skin inflammation, mast cell infiltration, IgE production, and inflammatory cytokines such as IL6 and TNF-α [19]. Although the sources of MSC isolation were different, our findings are consistent with those of previous studies on the immunomodulatory effects of MSC-based therapies in AD mouse models. In the present study, to explore and compare the therapeutic effects between primed T-MSCs and naïve T-MSCs, we subcutaneously injected primed T-MSCs or naïve T-MSCs in the DNFB-induced AD mouse model. Interestingly, primed T-MSCs suppress atopic inflammation more than naïve T-MSCs through the regulation of mast cell infiltration, Th2 cytokines, and IgE production in the mouse model of AD.

B cells play an essential role in the immune system, and their dysfunction can lead to various chronic inflammatory or autoimmune diseases [20]. Although the immunosuppressive properties of MSC-based therapies are not fully understood, increasing evidence has revealed changes in B cell function after MSC transplantation. Previous studies have confirmed the inhibitory effects of MSCs on B cell proliferation, maturation, and IgE production, and the effects of MSCs on B cells have also been shown to be attenuated by the addition of a selective COX-2 inhibitor [14,21,22]. Moreover, MSCs may induce regulatory B cells to secrete IL-10, which promotes the immunosuppressive function of B cells [23,24]. In our previous study, primed T-MSCs expressing CD40 showed immunomodulatory effects via Th1 and Th2 immune responses [16]. Consistent with this, in the present study, we found that the proliferation of B cells and the proportion of IgE-producing B cells were significantly suppressed by primed T-MSCs expressing CD40. MSCs may regulate immune responses via cell-to-cell contact; thus, their downstream pathways could be directly involved in B cell production, activation, and differentiation [25,26,27]. In line with this, in this study, we found that primed T-MSCs regulated B cell activation in a cell-to-cell contact-dependent manner.

One of the essential costimulatory molecules in the immune response is the receptor CD40, expressed on various cells such as B cells and antigen-presenting cells [28]. CD40 binds its ligand CD40L, transiently expressed on T cells and other non-immune cells during inflammatory conditions. CD40/CD40L interactions exert profound effects on various cell types, including B cells, dendritic cells, and endothelial cells. In B cells, CD40 signaling promotes germinal center formation, immunoglobulin (Ig) isotype switching, and memory B cell survival [29,30]. A previous study revealed that CD40+ cells in bone marrow-derived MSCs may be a key immune regulatory element in the bone marrow [31]. Our previous study demonstrated that CD40 may be a key molecule in the immunomodulatory capacity of primed T-MSCs [16]. Thus, to assess the critical role of CD40 as a regulator of B cell activation in primed T-MSCs, we knocked down CD40 expression in primed T-MSCs using a CD40-specific siRNA. CD40-knockdown primed T-MSCs significantly increased the activation and differentiation of B cells, suggesting that CD40 is a key molecule in primed T-MSCs for B cell regulation. However, the downstream mechanisms of CD40/CD40L in MSCs remain poorly understood. Thus, to examine the downstream mechanisms of CD40/CD40L, we examined the noncanonical and canonical NF-κB pathways in primed T-MSCs. We found that under B cell activation, the non-canonical NF-κB pathway was activated more dramatically than the canonical NF-κB in primed T-MSCs, and that CD40 expression on primed T-MSCs was also regulated by the non-canonical NF-κB pathway. However, our study also has limitations. Our method for the CD40 knockout is impossible to achieve a complete knockout and it induces only knockdown. Thus, this is a partial knockout method. Moreover, to confirm the findings of in vitro experiments, in vivo investigation using CD40 knockout mice should be needed.

In conclusion, our results showed that primed T-MSCs significantly improve the inflammatory status in an AD mouse model, and that this effect was mediated by the modulation of B cell-mediated inflammatory responses. Additionally, our data suggest that the regulatory effect of T-MSCs is dependent on CD40 expressed on primed T-MSCs through the non-canonical NF-κB pathway. Nevertheless, MSC-based therapies still face important challenges, including the type of stem cells used, the number of transplanted cells, pretreatment of cell products, relevant treatment targets, routes, and frequency of administration. Thus, additional research regarding these issues is necessary for our findings to be applied in practical clinical treatment.

## Data Availability

All data presented in this study are available in this article.
